# Design guidelines for limiting and eliminating virtual reality-induced symptoms and effects at work: a comprehensive, factor-oriented review

**DOI:** 10.3389/fpsyg.2023.1161932

**Published:** 2023-06-09

**Authors:** Alexis D. Souchet, Domitile Lourdeaux, Jean-Marie Burkhardt, Peter A. Hancock

**Affiliations:** ^1^Heudiasyc UMR 7253, Alliance Sorbonne Université, Université de Technologie de Compiègne, CNRS, Compiègne, France; ^2^Institute for Creative Technologies, University of Southern California, Los Angeles, CA, United States; ^3^IFSTTAR, University Gustave Eiffel and University of Paris, Paris, France; ^4^Department of Psychology, University of Central Florida, Orlando, FL, United States

**Keywords:** virtual reality, ergonomics, cybersickness, visual fatigue, muscle fatigue, acute stress, mental overload, work

## Abstract

Virtual reality (VR) can induce side effects known as virtual reality-induced symptoms and effects (VRISE). To address this concern, we identify a literature-based listing of these factors thought to influence VRISE with a focus on office work use. Using those, we recommend guidelines for VRISE amelioration intended for virtual environment creators and users. We identify five VRISE risks, focusing on short-term symptoms with their short-term effects. Three overall factor categories are considered: individual, hardware, and software. Over 90 factors may influence VRISE frequency and severity. We identify guidelines for each factor to help reduce VR side effects. To better reflect our confidence in those guidelines, we graded each with a level of evidence rating. Common factors occasionally influence different forms of VRISE. This can lead to confusion in the literature. General guidelines for using VR at work involve worker adaptation, such as limiting immersion times to between 20 and 30 min. These regimens involve taking regular breaks. Extra care is required for workers with special needs, neurodiversity, and gerontechnological concerns. In addition to following our guidelines, stakeholders should be aware that current head-mounted displays and virtual environments can continue to induce VRISE. While no single existing method fully alleviates VRISE, workers' health and safety must be monitored and safeguarded when VR is used at work.

## 1. Introduction

The COVID-19 pandemic conditions have accelerated the democratization of itinerant and remote work (Gajendran et al., 2021), making virtual reality (VR) an attractive alternative to support remote and collaborative office work (Ofek et al., 2020) and fostering the potential for its mass adoption (Grubert et al., 2018; Fereydooni and Walker, 2020; Knierim and Schmidt, 2020). While the potential benefits of VR have been widely reported in the literature, several authors (Keller and Colucci, 1998; Stanney et al., 1998; Sharples et al., 2008; Melzer et al., 2009; Fuchs, 2017, 2018; Souchet, 2020; Anses, 2021; Grassini and Laumann, 2021; Souchet et al., 2022) have stressed the necessity to address potential health and safety-related side effects of VR exposure. We focus specifically on office work use of VR.

Many terms have referred to such adversarial effects in the literature, most notably “cybersickness,” “VR sickness,” or “Simulator sickness.” In this study, we adopt the terms virtual reality-induced symptoms and effects (VRISE) introduced by Cobb et al. (1999) as it elicits a complete picture of the variety of VR side effects. VRISE initially encompasses cybersickness, postural instability, and other effects on psychomotor control, perceptual judgment, concentration, stress, and physical ergonomics (Cobb et al., 1999; Nichols, 1999; Nichols and Patel, 2002). Besides cybersickness, which is the most documented VRISE, the literature highlights four other undesired deleterious effects: visual fatigue, muscle fatigue, musculoskeletal discomfort, acute Stress, and mental overload. We propose to distinguish between cybersickness and visual fatigue. Indeed, cybersickness mostly refers to visually induced motion sickness that negatively impacts oculomotor function (Wang Y. et al., 2019). However, visual fatigue can occur without visually induced motion sickness (Souchet et al., 2022). Additionally, to health and safety concerns, the occurrence of VRISE can also induce a negative user experience (Somrak et al., 2019; Lavoie et al., 2020) and drastically impair performance in the task. For recent reviews of and in-depth discussions of VRISE, see, e.g., Ref Stanney et al. (2020b, 2021b), Howard and Van Zandt (2021), and Souchet et al. (2022).

Despite continuous improvements in the related technologies and the most recent innovations, the literature still provides evidence of VRISE with simulators and virtual environments. For example, Saredakis et al. (2020) found a mean dropout rate of 15.6% (min = 0%, max. = 100%) based on data reported in 44 empirical studies from the 55 selected for their systematic review of cybersickness and VR content impact with a head-mounted display (HMD). More generally, according to Stanney et al. (2021a) some side effects could be experienced even by more than 80% of VR users.

The research on VRISE has revealed that deleterious responses of users to virtual environment (VE) exposure vary widely depending on several factors, among which are the characteristics and capabilities of the users, the system (hardware/software) characteristics, and the implemented tasks to be performed with the VE. Unfortunately, no complete and holistic approaches to these different VRISE-related factors to be considered at the design and evaluation stages of VE development have been provided as far as we know. The literature provides some lists of factors specific to one single VRISE [e.g., for cybersickness, see Davis et al. (2014); LaViola et al. (2017)] or reports on a specific subset of factors that can influence VRISE. The latter include, for example, the visual fatigue caused by stereoscopy (Bando et al., 2012), cybersickness (Mittelstaedt, 2020; Howard and Van Zandt, 2021; Rebenitsch and Owen, 2021), and a panoply of other VRISE issues that could arise with VR usage (Chen et al., 2021). Factors are described, however, at various degrees of detail and completeness with no systematic wording consistency. Further limitations include that it is not always clear whether the claimed factors are grounded on empirical evidence, nor if they were identified in a VR context (Stanney et al., 2020b, 2021b; Howard and Van Zandt, 2021; Souchet et al., 2022). Further shortcomings in the current literature are related to the confounding effects of VRISE on other psychophysiological effects or among them, as recently emphasized (Kourtesis et al., 2019). One VRISE could influence another, but very few direct experimental proofs allow us to appreciate the magnitude of those influences (Alsuraykh et al., 2019; Mittelstaedt, 2020; Sepich et al., 2022; Souchet et al., 2022).

Developing the use of VR at work can result in increased exposure of the population to these multiple side effects and their impact on workers' health and safety (LaViola et al., 2017; Fuchs, 2018; Khakurel et al., 2018; Çöltekin et al., 2020; Olson et al., 2020; Anses, 2021; Ens et al., 2021). Such risks were featured in the European Agency for Safety and Health at Work warning (EU-OSHA, 2019). Thus, it is critical to examine and organize the current knowledge on the whole set of potential VRISE relevant to using VR in a work context. This knowledge includes evidence associated with the various factors involved in VRISE occurrence (e.g., individual, contextual, or technological) and design resources and solutions susceptible to avoiding these effects or at least decreasing their impact and likelihood. In particular, design guidelines and principles provide essential resources. They can be combined with and integrated with all user-centered design processes. Design guidelines and principles have an extended history in human–computer interaction to support user interface decisions, e.g., Smith and Mosier (1986). Design decisions take advantage of extant practical experiences, results from user studies, and applicable experimental findings to promote application consistency. As technology develops, such guidelines have been adapted for or explicitly defined in VR (Gabbard et al., 1999; Stanney et al., 2003b, 2021a; Burkhardt et al., 2006). Particular devices and/or their components have driven guidelines regarding VR dimensions such as haptics (Hale and Stanney, 2004), 3D interaction (LaViola et al., 2017), or HMD's application in general (Vi et al., 2019). Guidelines for domain-specific applications or user profiles such as a therapist user interface (Brinkman et al., 2010), VR games (Desurvire and Kreminski, 2018), VR in human neuroscience (Kourtesis et al., 2019, 2020), and psychology (Vasser and Aru, 2020) or assessments of elderly users (Shamsuddin et al., 2011) have also been proposed. However, existing works provide only a limited and restricted consideration of VRISE directly (Souchet et al., 2022).

In a previous contribution (Souchet et al., 2022), we focused on defining the current state of the art regarding VRISE, emphasizing theoretical aspects and merging existing literature to provide a list of factors believed to influence VRISE. Following this previous publication, this study aimed to report on and organize a comprehensive review of published design guidelines associated with the five short-term VRISE cybersickness (CYB), visual fatigue (VF), muscular fatigue (MF), acute stress (S), and mental overload (MO), focusing on workers and vocational contexts. To assure that our guidelines are practical, we sought to consider typical tasks that office workers would usually undertake using a PC, but in our case using VR. In addition, we want to organize this review so that it is easy to use and apply by researchers, designers, and work professionals. For that purpose, we have ordered existing knowledge by VRISE, type of factors, and potential factors that may impact VRISE. Assessing VRISE factors can further help identify and establish how users, apparatus, and virtual environments each contribute to VRISE occurrence.

Our study is organized as follows. First, we describe the general method we employed to select articles or written descriptions of each identified factor. Second, a concise definition, symptomology, and prevalence description are distilled for each VRISE. We have based these on existing reviews, systematic syntheses, and meta-analyses. Third, within each VRISE presentation, we point to Tables describing each factor, and guideline, distinguished by three characteristics: (1) individual, (2) hardware, and (3) software. Fourth, within each VRISE presentation, we promulgate general guidelines according to our presented synthesis of existing knowledge. Fifth, we discuss our summated results and explore their advantages and limitations. Sixth, tables that assemble and present descriptions and guidelines by factors regarding each short-term VRISE are displayed.

## 2. Methods

We conducted a literature search on journal and conference papers related to the five VRISE and published between January 2016 and mid-2021 partially (Primary Elements 5, 6, and 7 are not applied) applying the comprehensive review methodology stated in Ref (Stratton, 2016). The start date was selected because it corresponds to Oculus CV1's commercial release, delineating the moment when HMDs become more widely accessible for laboratories and other facilities and the public. Thus, it allows a targeted overview of contributions incorporating new-generation HMDs. HMDs are not the only devices allowing access to VR content (e.g., cave automatic virtual environment), but we focus on HMDs in the current review.

The review included the following search terms: (“Virtual Reality”) AND (“cybersickness” OR “visually induced motion sickness” OR “visual fatigue” OR “eyestrain” OR “muscle fatigue” OR “musculoskeletal discomfort” OR “stress” OR “acute stress” OR “cognitive load” OR “mental workload”) AND work AND (“meta-analysis” OR “systematic” OR “review”). This search was carried out on August 2021 on Scopus and Google Scholar.^1^

A first selection occurred based on titles and abstracts: We excluded those that did not refer to any of the five VRISE. Journal, conferences articles, and book chapters were included in this review if they were complete (i.e., includes a full paper, not just an abstract); the text was in English or French; the data were obtained from adults participants; the experimental tasks mainly were matching office-like tasks (text entry, document editing, reading, proofreading, gathering and processing data, creating graphs and data visualization (e.g., maps, plots), exploring and visually analyzing data, viewing several media (texts, images, videos, 3D objects), creating presentation materials, conducting meetings (public speaking), collaborating with other users in a shared VR environment. Additional papers anterior to 2016 were manually searched when no available review or meta-analysis was found regarding a VRISE or its related factors.

For each VRISE, we identified factors reported as associated with their occurrence and the proposed guidelines when provided. The definition and summary of the theories underlying the occurrence of each VRISE were made based on the most recent reviews or meta-analyses. Within each VRISE, we classified factors and guidelines into three (*1) individual, (2) hardware, and (3) software*, following LaViola (2000).

To better reflect our confidence in *those* guidelines, we graded each with a level of evidence based on Ackley et al. (2008) initially developed to assess nursing care evidences. Common factors occasionally influence different forms of VRISE. Hence, in those cases, crossing all VRISE can be important to envision what should be done to mitigate them.

As all empirical studies did not necessarily report guidelines, we translated the reported results as guidelines when it was the case. Hence, those guidelines are interpretations by the authors.

## 3. Results

### 3.1. Cybersickness

#### 3.1.1. Definition

Cybersickness has been defined as “an uncomfortable side effect experienced by users of immersive interfaces commonly used for Virtual Reality. It is associated with symptoms such as nausea, postural instability, disorientation, headaches, eyestrain, and tiredness” (Lavoie et al., 2020).

#### 3.1.2. Prevalence

Stanney et al. (2020b) have reported that at least one-third of users will experience cybersickness, with 5% of these participants presenting severe symptoms while using current HMDs generation, prevalence being almost necessarily contingent upon the technological state of the art (Somrak et al., 2019).

#### 3.1.3. Theoretical grounding

The sensory cue conflict proposition is widely accepted compared with competing theories (Lee and Choo, 2013; Stanney et al., 2020b). According to sensory cue conflict, cybersickness appears to occur because of visual–vestibular–proprioceptive conflicts (Roesler and McGaugh, 2019; Staresina and Wimber, 2019; Wong et al., 2019; Hirschle et al., 2020; Klier et al., 2020; Saredakis et al., 2020; Stanney et al., 2020b; Grassini and Laumann, 2021; Howard and Van Zandt, 2021). These inconsistencies are also called sensorimotor conflicts. However, the ecological theory (postural instability) also relies on extensive experimental results (Theorell et al., 2015; Aronsson et al., 2017; Stanney et al., 2020b). According to the ecological theory, humans primarily try to maintain postural stability. Hence, motion sickness expands with postural instability due to the novel environment and motion cues (Stanney et al., 2020b). Therefore, the cue conflict theory defends inconsistencies between perception systems, while the ecological theory defends postural instability, provoking motion sickness.

#### 3.1.4. Guidelines considering cybersickness factors

Rebenitsch and Owen (2021) have proposed 50 factors influencing cybersickness occurrence in VR. Unfortunately, in doing so, they do not limit to this relevant literature. However, they reuse Davis et al.'s (2014) list and align with the factors that Howard and Van Zandt (2021) noted. Mittelstaedt (2020) also proposed a synthesis. We selected Rebenitsch and Owen's (2014) factors list because it postulates more factors than other comparable publications. Each table lists one type or subtype of factor that could influence cybersickness:

- Individual factors related to experience with virtual environments and users' physical attributes are given in Table 1; general demographic factors and mental attributes are listed in Table 2.
- Hardware factors relating to screen are provided in Table 3, tracking in Table 4, rendering in Table 5, and non-visual feedback in Table 6.
- Software factors relating to movement in Table 7 and appearance and stabilizing information in Table 8.

**Table 1:** Guidelines for possible individual factors relating to experience with virtual environments (CYB_1 to 4) and users' physical attributes (CYB_5 to 9) influencing cybersickness.

**ID_factor Evidence level**	**Factors**	**Description**	**Guidelines**
CYB_1 *V*	Experience with a real-world task	Familiarity with tasks (real) in VR before being immersed seems to positively influence symptoms (Porcino et al., 2017; Howard and Van Zandt, 2021)	Acclimating users to tasks before immersing in VR could help reduce side effects occurrence (Howard and Van Zandt, 2021)
CYB_2 *V*	Experiences with a simulator (habituation)	Familiarity with immersive experiences drives users to report fewer symptoms (Howard and Van Zandt, 2021)	Acclimating users to immersive technologies before making them work in VR (Howard and Van Zandt, 2021; Szopa and Soares, 2021)
CYB_3 *VI*	Video gameplay	Users referred to as “gamers” are less susceptible to report high symptoms (Collaboration, 2015; Lanier et al., 2019; Kaplan et al., 2020; Szopa and Soares, 2021; Theresa Pöhlmann et al., 2021; Wang et al., 2021)	Encouraging potential users to play 3D video games to acclimate them to movements (Rebenitsch and Owen, 2021) on a screen they could encounter in VR
CYB_4 *III*	Duration	Cybersickness occurrence is linearly correlated with exposure duration (Duzmańska et al., 2018; Muthukrishna and Henrich, 2019; Rebenitsch and Owen, 2021)	Making short sessions at the beginning and increasing immersion time if users are building habituation. Cybersickness can arise after 5 min especially with very inducing contents (Anses, 2021)
CYB_5 *VI*	Eye dominance	“*Eye dominance refers to the preference to use one eye more than the fellow eye to accomplish a task*” (Ooi and He, 2020). It also seems to apply to binocular stimuli (Han et al., 2018). By stimulating both eyes equally or unequally, some peers think it can mitigate cybersickness (Meng et al., 2020; Hussain et al., 2021)	Eye-dominance-guided foveated rendering could help reduce non-necessary stimuli on the non-dominant eye, reducing symptoms occurrence (Meng et al., 2020; Hussain et al., 2021)
CYB_6 *VI*	Stereoscopic visual ability	See VF_2	See VF_2
CYB_7 *V*	Postural stability	Unstable (posture) users are more likely to become sick in line with Postural instability theory of cybersickness (Risi and Palmisano, 2019a; Stanney et al., 2020b). Although experimental results can sometimes contradict this prediction (Dennison and D'Zmura, 2017, 2018; Arcioni et al., 2019; Risi and Palmisano, 2019b; Kim J. et al., 2021; Litleskare, 2021)	Use questionnaires to determine if users are susceptible to postural instability to adapt exposure to him/her (Risi and Palmisano, 2019a; Stanney et al., 2020b; Howard and Van Zandt, 2021)
CYB_8 *VI*	History of headaches/migraines	Migraine (and Vestibular Migraine) history can predict part of cybersickness symptoms (Wang and Lewis, 2016; Paroz and Potter, 2017; Lim et al., 2018; Stanney et al., 2020a; MacArthur et al., 2021)	Determining if the user has a history of headaches or migraines to adapt exposure to him/her with questionnaires such as Visually Induced Motion Sickness Susceptibility Questionnaire (Keshavarz et al., 2021)
CYB_9 *VII*	Body mass index	The lower the body mass, the higher the reported symptoms (Stanney et al., 2003a, 2020a)	Determining user height and weight (questionnaire) and adapting exposure strategy (shorter duration, more pre-exposure before real work tasks) if more susceptible to present symptoms (Stanney et al., 2020a)

**Table 2:** Guidelines for possible general demographic factors (CYB_10 to 14) and mental attributes (CYB_15 to 17) influencing cybersickness.

**ID_factor Evidence level**	**Factors**	**Description**	**Guidelines**
CYB_10 *VII*	Age	Fifty years old and older are more susceptible to report cybersickness than younger people (Arns and Cerney, 2005; Petri et al., 2020; Kim H. et al., 2021). Children aged between 4 and 10 seem less susceptible than adults (Tychsen and Foeller, 2020), although they are practically not susceptible to using VR for work	Take extra care and expose for shorter time users of 50 years old and older to VR (Arns and Cerney, 2005; Petri et al., 2020; Kim H. et al., 2021)
CYB_11 *V*	Gender	Women are supposed to be more susceptible to cybersickness (Grassini and Laumann, 2020; Howard and Van Zandt, 2021). Although, there is no consensus because of contradictory results (Porcino et al., 2020a; MacArthur et al., 2021; Varmaghani et al., 2021). The observed difference is mainly explained by tuning lenses distance not matching womens' IPD (Stanney et al., 2020a)	Choosing HMDs allowing the widest tuning of lenses to match women's IPD and using eye tracking (or psychometric measures) to guide users when they tune the distance between lenses (Grassini and Laumann, 2020; Stanney et al., 2020a; Howard and Van Zandt, 2021)
CYB_12 *V*	Ethnicity	According to some studies (Klosterhalfen et al., 2005; Stanney et al., 2020a,b), Asians are more susceptible than African Americans and Caucasians	Be extra careful with ethnicity identified as being more susceptible to present side effects (Klosterhalfen et al., 2005; Stanney et al., 2020a,b)
CYB_13 *V*	Vision correction	Using glasses and/or contacts makes the user more susceptible to report cybersickness (Rebenitsch and Owen, 2014). However, contradictory results have been found (Rangelova et al., 2020)	Take extra care of users wearing lenses or glasses by encouraging to shorter immersion duration and more acclimation to simulators/3D video games (Rebenitsch and Owen, 2014; Howard and Van Zandt, 2021)
CYB_14 *V*	History of motion sickness	Prior history of motion sickness increase risks of cybersickness (Stanney et al., 2003a; Mittelstaedt et al., 2018; Mittelstaedt, 2020)	Determining if the user has a history of motion sickness to adapt exposure to him/her with questionnaires such as Visually Induced Motion Sickness Susceptibility Questionnaire (Keshavarz et al., 2021)
CYB_15 *VI*	Concentration level	Announced to influence cybersickness but without experimental data (Rangelova and Andre, 2019; Grassini et al., 2021; Rebenitsch and Owen, 2021)	Not clear in the cybersickness literature if “concentration” relates to attention abilities in general (Moran, 1763; Fawcett et al., 2015). Therefore, making sure that users can concentrate on tasks during immersion with eye tracking could help integrate this factor (Clay et al., 2019)
CYB_16 *VI*	Mental rotation ability	Sex differences have been raise (Parsons et al., 2004; Guzsvinecz et al., 2021), although contradicting results exist (Toth and Campbell, 2019). Mental rotation ability could be an unreliable factor affecting cybersickness (Mittelstaedt et al., 2019), although it is listed as possibly impacting symptoms (Stanney et al., 2020a)	Performing mental rotation tests (Shepard and Metzler, 1971). In VR paradigms exist (Csincsák, 2020) and hypothesizing that lower results would advocate for higher cybersickness risks
CYB_17 –	Perceptual style	Perceptual style influencing motion sickness is proposed in an old contribution (Barrett and Thornton, 1968). However, perceptual style is linked to learning style, criticized as a neuromyth (Willingham et al., 2015; Kirschner, 2017)	Since very little experimental proof and this factor might be linked to a neuromyth (Willingham et al., 2015; Kirschner, 2017), it can be ignored for now

**Table 3:** Guidelines for possible hardware factors relating to screen influencing cybersickness.

**ID_factor Evidence level**	**Screen factors**	**Description**	**Guidelines**
CYB_18 *IV*	Resolution/blur	The lower the resolution, the higher could be cybersickness (Palmisano et al., 2017). Although resolution could have a marginal impact (Caserman et al., 2021). Peripheral blurring showed encouraging results at mitigating cybersickness (Lin et al., 2020; Groth et al., 2021a,b)	Preferring HMDs with the highest resolutions—at the time—if possible cybersickness (Palmisano et al., 2017). Applying peripherical blur during movements (Groth et al., 2021a)
CYB_19 *V*	Horizontal and vertical field of view	The peripheral vision field is higher in females than males, increasing flicker likelihood (Davis et al., 2014; Chang et al., 2020; Stanney et al., 2020a; Teixeira and Palmisano, 2021)	Applying a field of view reduction (Groth et al., 2021a) and wider for women to reduce cybersickness during movement
CYB_20 *VI*	Weight of the display	See MF_3 and MF_4 We can hypothesize that displays' weight concurs to tiredness symptoms of cybersickness. However, it is pointed to as having minor effects on cybersickness (Rebenitsch and Owen, 2021)	Depending on HMD design, weight can be divided between various parts of users' heads. Using the lightest HMD might not be the best choice depending on straps (see MF_3 and MF_4). Allowing the user to do frequent breaks can help to recover (Chang et al., 2020)
CYB_21 *V*	Display type	According to Rebenitsch and Owen (Rebenitsch and Owen, 2016), HMD is the VR display type with which users report more cybersickness symptoms	Using HMD only if they are proved to be more efficient for a work task than another display type (Chang et al., 2020; Howard and Van Zandt, 2021)
CYB_22 *IV*	Lag variance	Tracking systems, graphical performance of PC or HMDs (standalone), and communication between hardware and software, in general, can cause latencies in displayed images or feedbacks (Chang et al., 2020; Stanney et al., 2020b). Those lags increase cybersickness symptoms (Rebenitsch and Owen, 2016; Palmisano et al., 2019)	On-screen (visual) latency should be inferior to 17–20 ms, although those values are debatable (Stauffert et al., 2020). Measuring constantly the latency of the virtual environment (Stauffert et al., 2021)

**Table 4:** Guidelines for possible hardware factors relating to tracking influencing cybersickness.

**ID_factor Evidence level**	**Tracking (hardware)**	**Description**	**Guidelines**
CYB_23 IV	Method of movement	This factor is possibly the most influencing cybersickness occurrence as objects locomotion in VR provokes vection. Vection and self-movement perception are affecting cybersickness symptoms (Keshavarz et al., 2014; Palmisano et al., 2017; Gallagher and Ferrè, 2018; Chang et al., 2020; Chardonnet et al., 2020; Descheneaux et al., 2020; Kemeny et al., 2020; Stanney et al., 2020b; Yildirim, 2020; Caserman et al., 2021; Fauville et al., 2021)	Several postures and interactions can be used in VR: sitting, standing, and walking (Bellgardt et al., 2017). However, sitting without virtual locomotion seems the most advantageous use of VR in our case (Zielasko and Riecke, 2020). If locomotion is necessary in the virtual environment, the best to reduce potential cybersickness, also relative to users' posture, are in order (Kemeny et al., 2020; Porcino et al., 2020a; Caserman et al., 2021): 1) Avoid continuous movements 2) Field of view reduction during movement 3) Teleportation, although depending on the virtual environment, can be inefficient (Clifton and Palmisano, 2020) 4) Adding “noise” to vestibular cues (Weech et al., 2020) 5) Using tracking of the entire body Depending on locomotion, the transition style can also impact usability (flying can be better than teleporting for spatial awareness) (Coburn et al., 2020)
CYB_24 V	Calibration	Poor calibration increases cybersickness symptoms as users' physical characteristics vary, while hardware allows limited match ranks between it and tracking devices (Davis et al., 2014). Poor calibration can cause delays, lags, and incongruent feedbacks in the virtual environment	Ensuring that tracking devices are correctly calibrated and work with each user (correct size, accurate focus, and correct alignment) (Davis et al., 2014). A checklist of what needs to be calibrated before VR use could help
CYB_25 V	Position tracking error	Head tracking gets worst linearly with use time (Garcia-Agundez et al., 2017). In general, position tracking error can create poor stimuli, feedback, interactions with the virtual environment (Davis et al., 2014)	Testing apparatuses possible tracking errors, using an HMD adapted to the physical space or convertibly (Garcia-Agundez et al., 2017; Chang et al., 2020)
CYB_26 V	Tracking method	Part of last generation HMDs still depend on external trackers. Depending on the tracking method, the error rate can variate and impact interaction. Therefore, influencing other tracking factors and movements (Chang et al., 2020)	If locomotion needs to be very accurate, content creators should consider HMDs' tracking method since it impacts further other factors influencing cybersickness (Chang et al., 2020). Tracking should match a >60 Hz refresh rate (Davis et al., 2014)
CYB_27 VI	Head movements	Head rotation and translation movements can impact cybersickness (Palmisano et al., 2017, 2020). The more head movements, the more cybersickness risks, although some tolerance is possible (Kim J. et al., 2021). Head movements can be correlated with tasks and stimuli distance (depth) (Pöhlmann et al., 2021)	Allowing users to take a break in head movements during VR use (Kim J. et al., 2021)

**Table 5:** Guidelines for possible hardware factors relating to rendering influencing cybersickness.

**ID_factor Evidence level**	**Rendering (hardware)**	**Description**	**Guidelines**
CYB_28 *V*	Stereoscopic rendering	Stereoscopy seems to increase cybersickness symptoms (Isaza et al., 2019; Palmisano et al., 2019). It collaborates with vection (Chang et al., 2020)	Using bi-ocular images (same image for each eye), not stereoscopy (Isaza et al., 2019; Palmisano et al., 2019)
CYB_29 *V*	Inter-pupillary distance	The HMD's lenses range of adjustment mismatch user's IPD (Stanney et al., 2020a). Women are more susceptible to cybersickness because of the impossibility of matching lens distance with IPD. Also see VF_4	Stanney et al. (2020a) call for HMD adjustable lenses matching more than 99% of IPDs in the general population, ranging from about 50 to 77 mm. Preferring HMDs with the widest lenses distance tuning. Measuring users' IPD with psychophysical tests or eye tracking to help them tuning HMDs correctly
CYB_30 *V*	Screen distance to the eye	In an HMD, screen distance is constant, very close to users' eyes, and stimuli are physically projected at a longer distance with lenses (Watson and Hodges, 1995). Accommodation occurs on the screen, while vergence occurs on objects at various depths (Souchet, 2020). The closer the screen or projected screen, the harder for the eyes to accommodate without diplopia. Also see VF_3	Using HMDs with lenses projecting images at a comfortable distance: < 2 m (Patterson, 2009) Applying “on-screen” parallaxes to alleviate vergence-accommodation conflict (Fuchs, 2017) Not displaying stereoscopy (Souchet, 2020)
CYB_31 *V*	Update rate	Users are in constant interaction with the virtual environment by providing inputs that induce feedback. That feedback occurs by updating the current virtual environment's stimuli (objects, movements, sounds…). A slow update rate can create incongruence between users' inputs and the virtual environment's feedback (Davis et al., 2014). Current HMD generation allows an images update rate of 60–144 Hz. Update (or refresh) rate could have a minor impact on cybersickness (Rebenitsch and Owen, 2017, 2021; Porcino et al., 2020b; Saredakis et al., 2020; Caserman et al., 2021)	Current HMDs are usually allowing a 90 Hz image update rate Preferring HMDs with the highest update (refresh) rate if possible (Davis et al., 2014) Avoiding interactions requiring numerous changes and feedbacks in the virtual environment to reduce incongruence in synchronization between inputs and changes in the virtual environment (Davis et al., 2014)

**Table 6:** Guidelines for possible hardware factors relating to non-visual feedback influencing cybersickness.

**ID_factor Evidence level**	**Non-visual feedback (hardware)**	**Description**	**Guidelines**
CYB_32 *VI*	Type of haptic feedback	Haptic feedback allows adding acceleration cues, therefore, movement information (Porcino et al., 2020b). Adding haptic stimuli doesn't always positively affect cybersickness (Plouzeau et al., 2017; Gonçalves et al., 2020). But it also appears that it can reduce cybersickness (Liu et al., 2019)	Adding haptic feedback (e. g., vibrations) related to movement could alleviate cybersickness (Plouzeau et al., 2017; Gonçalves et al., 2020)
CYB_33 *VII*	Ambient temperature	HMDs themselves produce heat and can lead to thermal discomfort (Wang Z. et al., 2019). It can impact eyes tear films (Turnbull et al., 2019). Ambient temperature doesn't always impact cybersickness symptoms (Saeidi et al., 2021). Devices to stimuli thermoception exist (Han P. H. et al., 2017; Günther et al., 2020; Lee et al., 2020; Liu et al., 2021b). Airflow seems to reduce cybersickness (D'Amour et al., 2017; Harrington et al., 2019) but not always (Paroz and Potter, 2018)	No clear guidelines can be drawn from the literature on the most suitable temperature for VR use. Thermoception depends on what part of the body is at stake (Kim et al., 2017; Viana and Voets, 2020) and relative temperative adaptation duration depending on inside and outside delta. Devices stimulating users' thermoception could generalize. Ideal ambient temperature for VR use is not clear. 37°C is the average human internal temperature. Stimuli that increase to fever temperature could participate in cybersickness symptoms. We can hypothesize that wearing an HMD can get uncomfortable, mainly because the device also produces heat while functioning
CYB_34 *VII*	Olfactory feedback	Smell doesn't always impact cybersickness, whether positively or negatively (Narciso et al., 2019). But it can reduce symptoms (Ranasinghe et al., 2020)	Olfactory stimuli could help drive visual attention, impacting movement perception (Tsai et al., 2021) Researches still need to address how olfactive stimuli can influence or not cybersickness
CYB_35 *VII*	Audio feedback	Audio-visual mismatches could participate in cybersickness, although no clear proof exists (Siddig et al., 2019; Widyanti and Hafizhah, 2021). Therefore, audio feedback needs to be coherent as it could influence cybersickness. However, few contributions address this issue	Create matching audio-visual cues in virtual environments to allow spatial congruency and coherent movement perception (Stanney et al., 2020b)

**Table 7:** Guidelines for possible software factors relating to movement influencing cybersickness.

**ID_factor Evidence level**	**Movement (software)**	**Description**	**Guidelines**
CYB_36 *IV*	Rate of linear rotational acceleration	Linear rotational acceleration influences cybersickness (Kim et al., 2017; Paroz and Potter, 2018; Harrington et al., 2019; Clifton and Palmisano, 2020; Kemeny et al., 2020; Weech et al., 2020; Kirollos and Herdman, 2021)	Use a low rate of linear rotational acceleration, and if higher are necessary (Kemeny et al., 2020; Viana and Voets, 2020), introduce them gradually
CYB_37 *V*	Self-movement speed and rotation	Proprioception is the sensation of body position and movement (Narciso et al., 2019). Whether the user is moving or not, while the visual feedback induces movement, the self-movement speed and rotation can mismatch those feedback (Lin et al., 2020; Ranasinghe et al., 2020). It mainly seems that humans have preattentive processing of visual self-motion information (Tsai et al., 2021) and gaze stabilization strategy during self-motion to control our body (Siddig et al., 2019). User's representation (avatar) can influence proprioception depending if legs and arms are present in 1st-person perspective or if the user is represented in 3rd-person perspective (Kemeny et al., 2020; Kim J. et al., 2020; Terenzi and Zaal, 2020; Widyanti and Hafizhah, 2021) See also CYB_38	Encourage low self-movement speed and rotation by users. When walking in VR, 1.4 m/s is recommended (Paroz and Potter, 2018; Kemeny et al., 2020). These factors depend on locomotion technique (Rebenitsch and Owen, 2016; Boletsis, 2017; Plouzeau et al., 2018; Tuthill and Azim, 2018; Tian et al., 2020; Paik et al., 2021; Rantala et al., 2021) Since few investigations have been conducted in office-like work situations on which avatar's characteristics are the most suitable, the only guideline would be to allow the user to choose between 1st-person perspective or 3rd-person perspective
CYB_38 *V*	Vection	Four competing definitions of vection exist. We align with the definition of vection, stating it is: “*a visually mediated subjective experience of self-motion*” (Palmisano et al., 2015; Kim and Park, 2020). Vection could be influenced by cognitive factors and individual traits (field dependence and depersonalization) (Schmitt et al., 2021). Some results point that strong vection can lead to reduced cybersickness (Fawcett et al., 2015; D'Amour et al., 2021). Therefore, vection is seen as a possible way to alleviate cybersickness (Stanney et al., 2020b). Vection doesn't seem causal to visually induced motion sickness (Kuiper et al., 2019; Chow et al., 2021). A large Field of view can impact vection (de Winkel et al., 2018; van der Veer et al., 2019). See CYB_23 and CYB_37	See CYB_23 and CYB_37
CYB_39 *VI*	Altitude above terrain	Manipulating view height can impact body parts perception (Widyanti and Hafizhah, 2021)	Matching user's real height in the virtual environment and feedback adapted to virtual terrain variation (Widyanti and Hafizhah, 2021)
CYB_40 *VI*	Degree of control	Uncontrolled movements could influence cybersickness as users' can't predict the environment and resulting self-motion (Nesbitt and Nalivaiko, 2018; Evin et al., 2020; Weech et al., 2020). However, when tested directly as a cybersickness factor, the degree of control doesn't always have influence (Matsuda et al., 2021; Shi et al., 2021)	Avoiding uncontrolled movements (Matsuda et al., 2021; Shi et al., 2021)

**Table 8:** Guidelines for possible software factors relating to appearance (CYB_41 to 46) and stabilizing information (CYB_47 to 50) influencing cybersickness.

**ID_factor Evidence level**	**Factors**	**Description**	**Guidelines**
CYB_41 *IV*	Screen luminance	See VF_6	See VF_6
CYB_42 *IV*	Color	See VF_14	See VF_14
CYB_43 *VI*	Contrast	High contrast levels could lead to higher cybersickness symptoms (Zhang et al., 2010; Kemeny et al., 2020; Campos et al., 2021)	Selecting HMDs depending on their screen technology (OLED, LCD) allowing the best contrasts to control optical variables of the virtual environment. Trying to display low contracts (Campos et al., 2021)
CYB_44 *V*	Scene content or scene complexity	Adding complexity (more visual cues) could drive higher cybersickness symptoms, impacting motion perception (Allue et al., 2016; Porcino et al., 2017; Hu et al., 2019; Islam et al., 2020)	High content variation and complexity should be avoided (Porcino et al., 2017; Hu et al., 2019; Islam et al., 2020). Minimalist interfaces could help at reducing cybersickness symptoms
CYB_45 *VI*	Global visual flow	Visual flow influences walking speed (Mohler et al., 2007; Salinas et al., 2017). During walking, the velocity of visual self-motion feedback seems to impact gait (Janeh et al., 2017). Globally, navigation speed influences symptoms (So et al., 2001; Kwok et al., 2018). Optical flow can also influence head displacement (Fujimoto and Ashida, 2020). Globally, humans seem more nauseous when watching intermittently moving and static visual objects (Chang et al., 2020). Sensitivity to motion parallax cues drives more sensitivity to cybersickness (Fulvio and Rokers, 2021). In HMDs, FoV also influence the amount of visual stimuli user perceive, see CYB_19	Use low locomotion speed. When visual flow reproduces walking stimuli, starting at a 5 m/s speed could activate cybersickness (So et al., 2001)
CYB_46 *IV*	Orientation cues	The direction of visual flow influences cybersickness (moving forward induce more cybersickness than moving backward) (Gavgani et al., 2017). Globally, users can be disoriented in VR as conflicting visual and vestibular cues are displayed (Coburn et al., 2020; Palmisano et al., 2020; Porcino et al., 2020a; Tian et al., 2020; Yildirim, 2020; Chang et al., 2021) Since head tracking provides most orientation cues, poor tracking can induce mismatching cues, see CYB_25	Adding a visual cue in the virtual environment as a reference (both body representation and surrounding objects) (Funk et al., 2019; Petri et al., 2020). However, more orientation cues (realism) can drive more cybersickness (Rebenitsch and Owen, 2016). Allowing users to choose the avatar's perspective, viewing distance, and preview where they will land if teleporting (Cmentowski et al., 2019; Zhang et al., 2020a). Allowing users to choose the locomotion technique
CYB_47 *IV*	Focus areas	Focus areas outside the central vision can participate in cybersickness as peripheral vision is more sensitive to flicker (Descheneaux et al., 2020) See also CYB_11	Content should focus on users' central vision and near peripheral corresponding to 30° eccentricity angle horizontally (Bhise, 2012; Hussain et al., 2020; Wu et al., 2021) Using foveated rendering or FOV restrictor (depending on eye tracking) (Adhanom et al., 2020) See also CYB_11
CYB_48 *VI*	The ratio of virtual to real world	Being static in the real world while moving in VR impacts cybersickness, and the more differences (ratio) between real and virtual cues, the more symptoms (Saredakis et al., 2020). Real-world reference to give fixed stabilization information can positively impact cybersickness while moving in VR (Chojecki et al., 2021) This ratio can also concern virtual object size and distance compared to reality	Putting a fixed virtual “object” corresponding to a real “object” as a reference point for locomotion, object size, and depth (Chojecki et al., 2021) See also CYB_49
CYB_49 *VI*	Independent visual backgrounds	Moving background induce cybersickness (Jeong et al., 2019; Oh and Lee, 2021)	Having a fixed background in the virtual environment (Hemmerich et al., 2020; Rebenitsch and Owen, 2021)
CYB_50 *IV*	Siting vs. standing	Standing rather than sitting increases the chances to provoke cybersickness (Merhi et al., 2007)	Several postures and interactions can be used in VR: sitting, standing, and walking (Bellgardt et al., 2017). Sitting without virtual locomotion seems the most advantageous use of VR at work (Zielasko and Riecke, 2020)

### 3.2. Visual fatigue

#### 3.2.1. Definition

Visual fatigue can be defined as: “*physiological strain or stress resulting from excessive exertion of the visual system*” (Somrak et al., 2019). Sheppard and Wolffsohn (2018) reference the list of symptoms identified by the *American Optometric Association*. These include eyestrain, headache, blurred vision, dry eyes, and pain in the neck and shoulders.

#### 3.2.2. Prevalence

Visual fatigue is already a significant issue in everyday work, with a large population at risk estimated at around 50% (Nesbitt and Nalivaiko, 2018). Close-up work on computer screens is an issue regarding dry eyes, ametropia, and accommodation or vergence mechanisms (Lackner, 2014). New-generation HMDs still continue to cause visual fatigue (Koohestani et al., 2019; Wang Y. et al., 2019; Descheneaux et al., 2020; Kemeny et al., 2020; Caserman et al., 2021; MacArthur et al., 2021) alongside visual discomfort (Lambooij and IJsselsteijn, 2009; Sheppard and Wolffsohn, 2018; Ang and Quarles, 2020; Descheneaux et al., 2020; Yildirim, 2020). HMDs seem to create higher visual fatigue than PC, tablets, or smartphones (Souchet et al., 2018; Hirota et al., 2019; Descheneaux et al., 2020; Hirzle et al., 2020). However, as HMDs could summate with other screen usages, more prolonged exposure to screens, in general, leads to increasingly negative symptoms on the visual system (Souchet et al., 2019).

#### 3.2.3. Guidelines considering visual fatigue factors

Fourteen factors influence visual fatigue occurrence based on our update (Souchet et al., 2022) of Bando et al. (2012)'s list. Each table lists one type or subtype of factor that could influence visual fatigue:

- Individual and hardware factors influencing visual fatigue are shown in Table 9.
- Software factors influencing visual fatigue are provided in Table 10.

**Table 9:** Guidelines for possible individual (VF_1 to 3) and hardware (VF_4 to 7) factors influencing visual fatigue.

**ID_factor Evidence level**	**Factor**	**Description**	**Guidelines**
VF_1 *VI*	Age	Visual acuity seems to drop starting at 55–59 years (Radner and Benesch, 2019). Accommodation decreases with age, and around 40 people present presbyopia (Charman, 2008; Lambooij et al., 2009). Precision abilities of stereopsis diminish with increasing age (Schubert et al., 2016). Stereoscopic acuity decreases with increasing age (Zaroff et al., 2003). Pupil diameter decreases with increasing age, especially at low luminance (Guillon et al., 2016). Tear production decreases with age (Blehm et al., 2005) and dry eyes symptoms increase with age (Ding and Sullivan, 2012; Coles-Brennan et al., 2019; Tellefsen Nøland et al., 2021). Contradicting results show an impact of age on visual fatigue: it decreases with increasing age (Larese Filon et al., 2019) similar within age groups (Sánchez-Brau et al., 2020; Lin et al., 2021) increases with increasing age (Ranasinghe et al., 2016)	At 40 and more, visual functions seem to decrease. Therefore, this population could be more at risk. However, younger (under 40) seem to be more subject to visual fatigue Taking breaks (Chang et al., 2020)
VF_2 *III*	Stereoscopic visual ability (stereo-blindness)	Part of the population is “stereo-blind.” These individuals are missing or have immeasurable binocular depth perception. The proportion of concerned individuals varies according to tested populations and measurement conditions from 2.2% to 32% (Lambooij et al., 2009; Bosten et al., 2015; Hess et al., 2015). Poor stereo acuity drives higher visual fatigue (Ramadan and Alhaag, 2018)	Test users' stereoscopic ability before VR exposure with clinical tests that can also be implemented directly in VR (Piano et al., 2016; O'Connor and Tidbury, 2018; Jeon and Choi, 2019; Kara et al., 2020; Cárdenas-Delgado et al., 2021): normal stereoscopic acuity is 20 arc seconds (Steinman et al., 2014), 50 arc seconds to 400 arc seconds can be considered as poor stereoscopic acuity (Deepa et al., 2019) If users are stereoblind or have very low ability, don't display stereoscopic images
VF_3 *III*	Vergence-accommodation conflict	Stereoscopy induces the vergence-accommodation conflict (Ukai and Howarth, 2008; Bando et al., 2012). This conflict also arises with HMDs and provokes visual fatigue (Souchet et al., 2018, 2021; Yuan et al., 2018; Matsuura, 2019)	Don't display stereoscopic images as benefits at displaying them are not always obvious (Souchet et al., 2018; Saracini et al., 2020). Display biocular images. Avoid negative parallaxes if you need to use stereoscopy (Liu et al., 2021a). Ensure that disparity is constant (not changing all the time) (Speranza et al., 2006; Cai et al., 2017; Jacobs et al., 2019; Shen et al., 2019; Souchet et al., 2019) Make sure that virtual objects appear “on screen” (close to null disparity) to make vergence closer to accommodation (Fuchs, 2017). For other devices displaying stereoscopy, a viewing distance of 2 m or more are advised (Patterson, 2009): in HMDs, it advocates for objects in stereoscopy to be 2 m from the viewer Create a region of interest focus, applying blur on regions outside of interest (Carnegie and Rhee, 2015; Porcino et al., 2020b; Caputo et al., 2021) Try to make accommodation matching vergence with eye tracking (Hasnain et al., 2019)
VF_4 *IV*	Optical misalignment (between HMD lenses and eyes)	Lenses not matching user IPD provokes visual fatigue (Hibbard et al., 2020; Stanney et al., 2020a; Wang X. M. et al., 2020)	Choosing HMDs allowing the widest tuning of lenses to match the user's IPD (Stanney et al., 2020a; Wang X. M. et al., 2020). Measure IPD to guide users when they tune the distance between lenses (eye tracking can help) (Chang et al., 2020; Hibbard et al., 2020)
VF_5 *IV*	Geometrical distortion (especially for 360° video when acquisition mismatches display)	Geometrical distortions (in position, shape, color, brightness, camera misalignment) induce visual fatigue (Bando et al., 2012; Gao et al., 2018; Xia et al., 2019; Hwang and Peli, 2020). Viewing angles can impact visual discomfort in HMDs (Iskander et al., 2019; Ha et al., 2021)	Check for any geometrical distortion and correct it (Jones et al., 2015; Gao et al., 2018; Scarfe and Glennerster, 2019)
VF_6 *IV*	Luminance	In an HMD, lighting depends on screen luminance, and since the Field of view is limited, peripherical vision is dark (Lin C. W. et al., 2020) The brighter the stimuli displayed, the higher visual fatigue (Wang et al., 2010; Benedetto et al., 2014; Cai et al., 2020; Erickson et al., 2020; Hamedani et al., 2020; Wang K. et al., 2020) Luminance contrasts between the display and the surrounding induce visual fatigue (Leccese et al., 2021) Pupil dilations can result in an enhanced perceptual experience of brightness (Sulutvedt et al., 2021)	For reading in an office, standards for room lighting are set between 500 and 750 lx (Liu T. et al., 2017) Using a computer display screen at night (from 18:00 to 23:00), Xie et al. (2021) advise setting screen luminance at 28cd/m^2^ (5%screen brightness) and a (text-background) luminance contrast not lower than 0.725 Zhou Y. et al. (2021) argue for ambient illuminance and screen luminance levels in the range of 13.08–62.16 lx and 20.63–75.15 cd/m^2^. For instance, Oculus rift DK2 can reach a maximum of 94 cd/m^2^. 28 cd/m^2^ seems the most comfortable in HMDs (Ha et al., 2017)
			Luminance between scenes in HMDs should be harmonized to avoid very different brightness leading to higher discomfort (Ha et al., 2020) Apply dark mode with compensated luminous intensity (Vasylevska et al., 2019) Calibration of HMDs' screens (Toscani et al., 2019) Patterson (2009) advise interocular luminance differences and interocular contrast differences at less than 25%
VF_7 *VII*	Blue light (range from 400 to 490 nm)	Blue light implies less accommodation (Panke et al., 2019). It can impact (Anses, 2019) myopia (positive or negative) and dry eye syndrome It induces visual fatigue (Rabin et al., 2020; Zhang et al., 2020b). 480 to 490 nm disturb circadian rhythms during the evening, impacting sleep (Wahl et al., 2019), making users more susceptible to visual fatigue (Munsamy and Chetty, 2020)	Reducing blue light with a filter (Chiu and Liu, 2020). Although proofs of efficiency are still missing, blue-blocking lenses could be a solution (Singh et al., 2021; Vagge et al., 2021)

**Table 10:** Guidelines for possible software factors influencing visual fatigue.

**ID_factor Evidence level**	**Software**	**Description**	**Guidelines**
VF_8 *III*	Duration of display use	The longer HMD use, the higher visual fatigue (Yuan et al., 2018; Yue et al., 2018; Guo et al., 2019, 2020; Szpak et al., 2020; Marshev et al., 2021)	Visual fatigue symptoms can start after 10 min of use. About 20 min will induce visual fatigue. Therefore, breaks might occur every 15 min to prevent visual fatigue (Yuan et al., 2018; Yue et al., 2018; Chang et al., 2020)
VF_9 *IV*	Binocular disparity (possible and comfortable fusion)	High disparity can be fused without diplopia, but high disparity induces visual fatigue (Shibata et al., 2011; Patterson, 2015; Fuchs, 2017). Negative parallaxes lead to higher visual fatigue than positive (Sun et al., 2020)	Shibata et al. (2011) assume that the maximum and minimum relative distance of the comfort zone is between 0.8 and 0.3 D Apply ±1.0° disparity to avoid visual fatigue (Bando et al., 2012; Matsuura, 2019; Hibbard et al., 2020) However, according to Patterson (Patterson, 2009), fusion is possible from 80 arc minutes for high spatial frequencies and up to 8° for low spatial frequencies images
VF_10 *IV*	Motion parallax	Moving objects can induce more visual discomfort (Speranza et al., 2006). The more dynamism in videos, the more visual fatigue (Kweon et al., 2018). Vertical parallax induces visual fatigue (Sugita et al., 2019). However, motion parallax from head movement reduces visual discomfort (Kongsilp and Dailey, 2017) See also CYB_37 and CYB_45	Prefer slow-motion parallax cues in the virtual environment and avoid discontinuity (Speranza et al., 2006; Kweon et al., 2018; Sugita et al., 2019)
VF_11 *VII*	Texture gradients	Conflicting texture gradient could lead to more visual fatigue as those cues play a role in stereopsis (Lambooij et al., 2009; Su et al., 2018). Too sharp textures, when supposed to be far from the user, would be “unnatural” depth cues. Texture gradients can also inform about object orientation (Leroy, 2016), and if conflicting with other orientation cues and motion, it could participate in visual fatigue See also VF_10 and CY_46	When textures are determinant depth cues, make sure to reproduce gradients close to real visual perception to give orientation information (Leroy, 2016) See also VF_10 and CY_46
VF_12 *IV*	Occlusion	Objects hiding part of another will make it appear as “closer” to the viewer. If the object is supposed to be behind has other depths cues that make it closer, it could influence visual fatigue (Pietroszek, 2015; Leroy, 2016). The cues are ambiguous. When stereoscopy is displayed, since FoV in HMDs is limited, objects with negative parallax would be partially “cut” by limited FoV	Make sure to avoid ambiguous occlusion. Reducing the number of 3D objects can help. Reducing overlapping objects can help (Sidenmark et al., 2020), especially when you are supposed to reach and touch this object (Yu et al., 2021) Make sure that objects with stereoscopic cues are mainly located in the central vision
VF_13 *IV*	Blur	Blur can drive vergence and accommodation (Lambooij et al., 2009; Sweeney et al., 2014). Therefore, blurring objects where the visual system is supposed to rely on vergence and accommodation cues could lead to more symptoms	Apply blur in images carefully, not on objects of interest but on other objects in the scene to avoid driving unwanted accommodation (Lambooij et al., 2009; Sweeney et al., 2014) Also see CYB_18
VF_14 *IV*	Colors	The more frequent color changes, the higher visual fatigue (Kim et al., 2016) Color temperature seems to impact visual fatigue (Wang K. et al., 2020) Stereoscopic acuity can increase with increasing color discrimination ability (Koctekin et al., 2020) Color also has a link with luminance: see VF_6 and VF_7	Avoid highly changing colors Avoid highly saturated colors (Kim et al., 2016) Prefer low luminance colors See VF_6 and VF_7

Factors inducing visual fatigue are not, in most cases, the central focus of peers for reducing VRISE. Therefore, further research is recommended in order to draw more precise and quantified guidelines.

### 3.3. Muscle fatigue and musculoskeletal discomfort

#### 3.3.1. Definition

Muscle fatigue has been defined as an: “*exercise-induced reduction in the ability of a muscle or muscle group to generate maximal force or power*” (Yoon et al., 2020). Muscle fatigue frequently arises with screen work (Souchet et al., 2021).

#### 3.3.2. Prevalence

Repetitions of excessive muscular loads can lead to musculoskeletal disorders and are the most common (almost 24% of EU workers) work-related problem in Europe (Cho et al., 2017). Neck, shoulder, forearm, and hands pain as well as upper and low back pain, prove to be the primary disorders associated with office work (Guo et al., 2017, 2019; Han J. et al., 2017; Bracq et al., 2019). Sitting while performing computer work can be associated with short-term adverse effects, such as physical discomfort (Yu X. et al., 2018). Symptoms associated with prolonged use of computers are neck and wrist pain as well as backache (Zhang et al., 2020c). Such symptoms are likely to also arise in VR. However, the majority of the associated literature concerns sports activity and is relatively less concerning office work tasks. Many experiments on muscle fatigue and/or musculoskeletal discomfort are assessed primarily using smartphones, tablets, and computer screens. Rarely do these employ HMDs, although the trend is changing.

#### 3.3.3. Guidelines considering muscle fatigue and musculoskeletal discomfort factors

Fifteen factors have been identified (Souchet et al., 2022) as influencing muscle fatigue and musculoskeletal discomfort frequency of occurrence based on the current synthesis of existent work. Each table lists one type, or subtype, of factor that may influence muscle fatigue and musculoskeletal discomfort:

- Individual and Hardware factors influencing muscle fatigue and musculoskeletal discomfort are provided in Table 11.
- Software factors influencing muscle fatigue and musculoskeletal discomfort are described in Table 12.

**Table 11:** Guidelines for individual (MF_1 and 2) and hardware (MF_3 to 7) factors influencing muscle fatigue.

**ID_factor Evidence level**	**Factor**	**Description**	**Guidelines**
MF_1 *VI*	Age	Age as a muscle fatigue factor is unclear due to the lack of relevant data (Speed et al., 2018; Mahdavi et al., 2020) Older (≥55) persons have a stronger resistance to muscle fatigue during sustained and intermittent isometric contractions than younger persons (Smith and Mosier, 1986; Gabbard et al., 1999; LaViola, 2000; Nichols and Patel, 2002; Stanney et al., 2003b, 2020b, 2021a,b; Hale and Stanney, 2004; Burkhardt et al., 2006; Avin and Frey Law, 2011; Bando et al., 2012; Davis et al., 2014; LaViola et al., 2017; Khakurel et al., 2018; Alsuraykh et al., 2019; EU-OSHA, 2019; Kourtesis et al., 2019; Vi et al., 2019; Wang Y. et al., 2019; Çöltekin et al., 2020; Mittelstaedt, 2020; Olson et al., 2020; Saredakis et al., 2020; Chen et al., 2021; Ens et al., 2021; Howard and Van Zandt, 2021; Rebenitsch and Owen, 2021; Sepich et al., 2022). Shoulder abduction shows similar results (Collins and O'Sullivan, 2018). Aging doesn't rime with muscle strength loss (Kenny et al., 2016). Globally, older adults and males are more resistant to fatigue than adults and females (Wan et al., 2017). However, exercise performance decreases with aging, consequent to lower tolerance to peripheral fatigue (Zarzissi et al., 2020). Concurrent cognitive demand reduces more older adults' endurance (handgrip) than youngers' (Shortz and Mehta, 2017). Older workers perform less high-intensity physical activity than younger workers after work-related fatigue (Bláfoss et al., 2019). Older office workers also report higher general pain (Shariat et al., 2018) Bimanual coordination performance with imposed speed for task complexion seems more complex for older (Lambooij and IJsselsteijn, 2009; Sheppard and Wolffsohn, 2018; Souchet et al., 2018; Ang and Quarles, 2020; Hirzle et al., 2020; Yildirim, 2020; Caserman et al., 2021; MacArthur et al., 2021) people (Roman-Liu and Tokarski, 2021)	Avoid gestures that require strength for workers older than 55 (Roman-Liu and Tokarski, 2021) Consider that younger workers (Smith and Mosier, 1986; Gabbard et al., 1999; LaViola, 2000; Nichols and Patel, 2002; Stanney et al., 2003b, 2020b, 2021a,b; Hale and Stanney, 2004; Burkhardt et al., 2006; Bando et al., 2012; Davis et al., 2014; LaViola et al., 2017; Khakurel et al., 2018; Alsuraykh et al., 2019; EU-OSHA, 2019; Kourtesis et al., 2019; Vi et al., 2019; Wang Y. et al., 2019; Çöltekin et al., 2020; Mittelstaedt, 2020; Olson et al., 2020; Saredakis et al., 2020; Chen et al., 2021; Ens et al., 2021; Howard and Van Zandt, 2021; Rebenitsch and Owen, 2021; Sepich et al., 2022) might need shorter VR exposure due to fatigue resistance (Avin and Frey Law, 2011) Consider that too fatiguing VR use might reduce older workers' physical activity after work (and then impact general health) (Bláfoss et al., 2019)
MF_2 *VI*	Body mass index	Non-obese, overweight, and obese participants performing isometric contractions for shoulder flexion and trunk extension seems to have similar strength (Cavuoto et al., 2019). Body mass index doesn't seem to impact muscle fatigue (Russeng et al., 2020). However, low back pain severity (office workers) appears higher for individuals with a high body mass index (Shariat et al., 2018). Overweight/obese workers are more likely to present musculoskeletal pain and related symptoms in the shoulders (Moreira-Silva et al., 2013). Obese adults show shorter endurance duration than normal-weight adults only at lower intensities, larger and more postural muscles of the shoulder, and low back (Mehta and Cavuoto, 2017)	Users with obesity might be more likely to present muscle fatigue. According to this factor, adapting interactions for less tiring gestures could help prevent fatigue (Li G. et al., 2020)
MF_3 *IV*	HMD weight	HMD weight seems to add physical stress on the cheekbone and back of the head (Kim and Shin, 2018; Yan et al., 2019). Wearing a helmet during screen use induces neck pain (Le et al., 2021)	Chose the lighter HMD, or try to alleviate weight (Kim and Shin, 2018; Yan et al., 2019)
MF_4 *VI*	Belts (attaching HMD to head)	The lower the number of belts, the high perceived physical s0tress on the neck because of HMD's weight (Song et al., 2019)	Choose HMDs with more belts and support (Song et al., 2019) Add extra belts on HMDs
MF_5 *VI*	Interaction devices	Users can interact with their head movements, bare hands or controllers, and “laser pointers (Pietroszek, 2018; Dombrowski et al., 2019; Lu et al., 2019)” Interactions requiring bimanual coordination can be challenging for older persons (Roman-Liu and Tokarski, 2021). Depending on controllers' weight, they can participate in muscle fatigue and impact task performance comparably to induced fatigue in Dupuis et al. (2021)	If the user gets tired of interaction, consider input amplification (Wentzel et al., 2020). Allowing controllers' (or any other interaction device) sensitivity control by the user (Dombrowski et al., 2019) Be careful with mid-air interactions for both hands and controllers When possible, don't make users having to use both hands or controllers at the same time
MF_6 *V*	Position tracking error	The optimal center of mass position of HMDs varies depending on a user's posture (Chihara and Seo, 2018; Ito et al., 2019; Sun et al., 2019). Therefore, position tracking error would lead user's to compensate head (and other body parts) posture, leading to muscle fatigue See also CYB_7 and CYB_25	Prevent position tracking errors See CYB_7 and CYB_25.
MF_7 *IV*	HMD resolution	Depending on the task, here proofreading, resolution can contribute to physical stress (Kim and Shin, 2018)	Choose the HMDs with the highest resolution Consider foveated rendering (Patney et al., 2016; Alexandrov and Chertopolokhov, 2021; Franke et al., 2021) See also CYB_18

**Table 12:** Guidelines for software factors influencing muscle fatigue.

**ID_factor Evidence level**	**Software**	**Description**	**Guidelines**
MF_8 *VI*	Duration of immersion	Mobile touch screen device use duration is associated with increased musculoskeletal discomfort (Toh et al., 2017; Zirek et al., 2020). Findings are similar for computer use: neck pain (Keown and Tuchin, 2018; Coenen et al., 2019). This can also apply to VR (Lee and Han, 2018; Li M. et al., 2020). Ten minutes of VR use can be enough to induce musculoskeletal discomfort (Arif et al., 2021)	Limit use duration (Sesboüé and Guincestre, 2006). Depending on the task (i.e., laparoscopic tasks), symptoms can appear after 15 min of use (Li M. et al., 2020) or even after 10 min (Arif et al., 2021) See also CYB_4 and VF_8
MF_9 *VI*	Object angle location	Shoulder flexion angle, neck flexion moment, muscle activities of the neck and shoulder, and excessive vertical target locations when interacting with targets at several angles in the 3D environment are likely to drive musculoskeletal discomfort (Kim and Shin, 2018; Penumudi et al., 2020). Texting on a smartphone can induce neck pain due to head angle (Lee et al., 2015). Depending on screen position angle, neck pain can arise (Szeto and Sham, 2008). Lowering the head too much seems to apply too much tension on the neck	Objects should be placed at the center (central vision), slightly to the right for those to interact with often, slightly below the horizontal line for keyboards, and slightly to the left for alerts or elements requiring users' refocussing (Zhou et al., 2021)
MF_10 *VI*	Gesture amplitude	Interaction gestures play a role in musculoskeletal discomfort depending on their amplitude (Li G. et al., 2020; Penumudi et al., 2020). Show that physical fatigue is higher in VR than the same task in reality (Ahmed et al., 2017)	Avoid interactions requiring too wide gestures (Li G. et al., 2020; Penumudi et al., 2020)
MF_11 *V*	Tasks repetition	Repetitive movements during screen work, especially keyboard and mouse, contribute to musculoskeletal symptoms (Coenen et al., 2019). On tablets, typing with a virtual keyboard can induce muscle fatigue (Lin M. I. B. et al., 2020). However, adaptation redistributing muscle demand could alleviate the strain of repetitive gestures (Pritchard et al., 2019)	Try to allow breaks from repetitive movements in interaction metaphors: e.g., hand, wrist, harm resting, shoulder and head resting loops by relying on eye tracking interactions (Majaranta, 2012; Majaranta and Bulling, 2014; Clay et al., 2019; Silva et al., 2019; Stanney et al., 2020b)
MF_12 *VI*	Head rotations required	HMD increases the head rotation during editing tasks compared to a computer screen, leading to neck discomfort (Kim and Shin, 2018). However, not moving the head (static neck flexion) for 10 min can induce neck pain (Mousavi-Khatir et al., 2018). Watching a video in VR could lead to not move the head for that long	Avoid continuous head rotations Avoid stationary heads for 10 min and more. As demonstrated by multiple monitors (PC), having multiple “regions of interest” can be more comfortable during work (Gallagher et al., 2021). However, using three screens showed a decrease in work performance (Iskander et al., 2018). In VR, giving users' freedom to choose the size and position of virtual displays can alleviate pain (Mcgill et al., 2020). Try to facilitate a neutral neck posture (Emerson et al., 2021). Concentrating interactions and feedback at the central vision might help
MF_13 *VI*	General posture	Prolonged smartphone use for texting induces rigid posture, increasing tension at the neck-shoulder level if the neck shows excessive flexion (D'Anna et al., 2021). Increased neck flexion (PC) angles drive higher activity in the upper trapezius muscle leading to neck and shoulder discomfort (Szeto et al., 2005)	Promote neutral posture (D'Anna et al., 2021) Avoid 3D object position, regions of interest that induce prolonged non-neutral postures (Davis et al., 2014; Shannon et al., 2019)
MF_14 *IV*	Sitting or standing	Quasi-standing work can provoke muscle fatigue (Wall et al., 2020). Walking seems more physically (neck) demanding than sitting when using smartphones (Flores-Cruz et al., 2019; Yoon et al., 2021). However, mobile device use drives lower extremity pain while sitting (Legan and Zupan, 2020). Sitting at work for hours provokes discomfort in all body regions over time (Baker et al., 2018; Waongenngarm et al., 2020)	Sitting could avoid too much muscle tension while performing office-like tasks in VR However, since prolonged sitting is also an issue, allowing the user to stand and/or walk while immerged could alleviate the downside of sitting (Ding et al., 2020)
MF_15 *IV*	Body parts representation and feedback (avatar)	Modifying postural/gesture feedbacks of a user's avatar in VR unconsciously drives the motor and muscular adjustments (Bourdin et al., 2019). This could lead the user to take postures or perform gestures leading to muscle fatigue	Create the most accurate feedback on the avatar's posture and gesture (Bourdin et al., 2019) Modify feedbacks to compensate user's non-neutral posture for reducing possible pain

Clear information about muscle fatigue and musculoskeletal discomfort associated with VR exposure remains problematically scarce. Only a few works using PC or smartphone provide coherent findings for HMDs. However, the body part mobilized here, the tension experienced with HMDs and the interaction device use might not be equivalent. Therefore, we sought to extrapolate information from screen uses to provide guidelines. Muscle fatigue and musculoskeletal discomfort depend on specific task characteristics (Alabdulkader, 2021), making generalization challenging to validate.

### 3.4. Acute stress

#### 3.4.1. Definition

Stress can be defined as a: “*condition in which an individual is aroused and made anxious by an uncontrollable aversive challenge*” (Gandevia, 2001). Acute stress represents a sudden or short time exposure incident (trauma, perceived threat, death of a loved one, job loss, etc.). Acute stresses are often juxtaposed with chronic stress, the latter being long-term effects (European Agency for Safety Health at Work, 2007; Coenen et al., 2019).

#### 3.4.2. Prevalence

Current knowledge does not allow us to define acute stress prevalence induced by VR use specifically outside of wild task-specific aspects and technostress. Introducing VR at work without the proper training could trigger techno-complexity (see S_3 in Table 13) and add up to all the other apparatus workers already use, which might trigger techno-overload (see S_4 in Table 13). One wide use of VR is remote meetings. Public speaking is stress-inducing, but it seems higher with VR (Helminen et al., 2019; Zimmer et al., 2019). Acute stress, in general, impairs executive functioning (Calik et al., 2022). According to LeBlanc (Eltayeb et al., 2009), stress diminishes the efficiency of selective attention (Heidarimoghadam et al., 2020; Frutiger and Borotkanics, 2021). Stress can also impair working memory and has been suggested to enhance memory consolidation (Baker et al., 2018). Stress has been observed to impair memory recall/retrieval (Borhany et al., 2018; Shannon et al., 2019). Therefore, we can assert that stress can act to impair work performance when fulfilling tasks in VR. And, of course, these effects are dependent on task typologies. At the occupational level, stress impacts workers' health, performance, and wellbeing (Sesboüé and Guincestre, 2006; Fink, 2016). It can lead to depressive symptoms (Fink, 2007), burnout symptoms (Shields et al., 2016), hypertension (LeBlanc, 2009), and/or type 2 diabetes mellitus (Bater and Jordan, 2020). Stressors can therefore impact VR adoption as they affect task completion novelty and the spectrum of tasks' typology.

**Table 13:** Guidelines for possible individual (S_1 and 2) and hardware (S_3 and 4) factors influencing acute stress.

**ID_factor Evidence level**	**Individual**	**Description**	**Guidelines**
S_1 *VII*	Age	Older workers appear more resilient to work-related stress (Hsu, 2019) Stress's negative impacts on memory performance are lower in older people (Hidalgo et al., 2019), although this doesn't reveal at the meta-analytic level (Shields et al., 2017). Similarly, older people seem less impacted by acute psychosocial stress (Vallejo et al., 2021) Stressful work is linked to slightly more sickness absence among older workers (Götz et al., 2018) Older workers or those with longer professional have greater difficulties with the increase of technological complexity for executing tasks (techno-complexity) (Marchiori et al., 2019)	Consider that younger workers could be more sensitive to induced stressors in VR working tasks (Hsu, 2019). Consider attributing fewer complex tasks or less socially stressful tasks to younger workers Older workers could be more susceptible to techno-stress (see also S_3 and S_4) (Marchiori et al., 2019). Consider attributing fewer complex tasks in VR to them
S_2 *II*	Body Mass Index (BMI)	A weak association between work stress (occupational level) and high BMI exists (Kouvonen et al., 2005; Magnusson Hanson et al., 2017). However, there are contradictory results (Myers et al., 2021) Obesogenic behaviors seem to induce higher perceived stress (Barrington, 2012) Psychosocial stress is positively associated with body mass index gain (Harding et al., 2014)	Consider that people with high BMI could be more sensitive to acute stress (Barrington, 2012; Harding et al., 2014)
S_3 *VII*	Techno-complexity	Techno-complexity defines the inherent quality of an ITC, which drives employees to feel that their computer skills are inadequate. Symptomology is poor concentration, irritability, memory disturbances as well as exhaustion. Since VR at the workplace is new for most workers, it is reasonable to presume it could lead to techno-complexity stress. Workers will have to constantly learn how to use this ICT (Tarafdar et al., 2019). But coping with VR induced Techno-complexity results in stress responses at the occupational level (Dragano and Lunau, 2020; Tarafdar et al., 2020; Weinert et al., 2020)	Train workers correctly to in-VR tasks, virtual environment's interactions, and interfaces to prevent techno-complexity (Tarafdar et al., 2019)
S_4 *VI*	Techno-overload	Techno-overload defines “*simultaneous, different streams of information that increase the pace and volume of work*” (Atanasoff and Venable, 2017). Inside this techno-overload, the “information overload” dimension (Nisafani et al., 2020) could apply in the context of data analyses in VR. Since VR is new for most workers and implies side effects, we can predict a high demand psychologically and physiologically (Atanasoff and Venable, 2017; Zhao et al., 2020)	Adapt information streams to lower-down techno-overload and consider cybersickness, visual fatigue, and muscle fatigue to make more difficult application tools, thus, inducing stress (Atanasoff and Venable, 2017; Zhao et al., 2020)

#### 3.4.3. Guidelines considering acute stress factors

Based upon our synthetic assessment of previous works, several factors are identified as influencing acute stress occurrence. We focused on nine of these (Souchet et al., 2022). They are couched in terms of office-like tasks. Each table lists one type of factor that influences acute stress:

- Individual and hardware factors influencing acute stress are shown in Table 13.
- Software factors influencing acute stress are given in Table 14.

**Table 14:** Guidelines for possible software factors influencing acute stress.

**ID_factor Evidence level**	**Software**	**Description**	**Guidelines**
S_5 *V*	Time pressure	Time pressure defines an (Denovan and Dagnall, 2019): “*insufficient time available to complete necessary tasks*.” This insufficient time available is an individual perception of the amount of time necessary to fulfill a task (Ordóñez et al., 2015). It is a challenging stressor that can be coped via extra efforts, leading to strain and exhaustion (Prem et al., 2018). Time pressure can impact performance negatively to resolve math problems (Caviola et al., 2017). E.g., time pressure during investigations reduces the number of hypotheses tackled (Alison et al., 2013; Kim S. et al., 2020). Time pressure can be a stressor that impairs performances (less with procedural tasks) (McCoy et al., 2014; Prasad et al., 2020). It can impact response time, e.g.to make a decision (Korporaal et al., 2020). But, defining a deadline has a positive effect on decision-making. Taking decisions under time pressure is usually presented as having a negative impact (Ordóñez et al., 2015). Time pressure negatively impacts performance (Arora et al., 2010) and decision-making (Modi et al., 2020) See also MO_1	Extend time to fulfill a task in VR to avoid inducing stress and impacting work performances (Arora et al., 2010; McCoy et al., 2014; Prasad et al., 2020) Evaluate specifically how time pressure can benefit specific tasks in VR See also MO_1
S_6 *IV*	Task difficulty	Task difficulty, which encompasses multitasking, negatively influences task performances as it requires a higher mental load (de Dreu et al., 2019; Bretonnier et al., 2020; Modi et al., 2020) Difficulty can also enhance task performance or not change performance (Song et al., 2011; Main et al., 2017) Difficulty can be seen as a stressor (Atchley et al., 2017) Seel also MO_2	Reduce task difficulty in VR to prevent acute stress or frustration via dynamic adaptations to the user or helping agents (Gupta et al., 2020; Halbig and Latoschik, 2021) Seel also MO_2
S_7 *II*	Public speaking	Workers can suffer from public speaking anxiety, common in the general population (Ebrahimi et al., 2019; Marcel, 2019; Gallego et al., 2022). Public speaking induces acute stress, even in healthy adults without public speaking anxiety, and is used with the Trier Social Stress Test (TSST) to study stress in-lab (Allen et al., 2017; Labuschagne et al., 2019; Narvaez Linares et al., 2020). Immersive virtual environments replicating the TSST showed a higher cortisol reactivity than non-immersive (Helminen et al., 2019; Zimmer et al., 2019). Stress-induced with the TSST can impact decision-making (Pabst et al., 2013). Meetings can be in English, like in multinational corporations where workers present foreign language anxiety (Aichhorn and Puck, 2017; Kelsen, 2019; Kim et al., 2019). Presentations in front of peers, debating and, decision making can be seen as a stressor. It applies in VR (Barreda-Ángeles et al., 2020)	Adapt audience feedback to lower down speaking anxiety (Allen et al., 2017; Labuschagne et al., 2019; Narvaez Linares et al., 2020) Provide help in the interface to lower stress at public speaking, especially when using a second language
S_8 *VI*	Exposure to distressing material	Distressing materials are stressors that can lead to secondary traumatic stress (Perez et al., 2010; Holt and Blevins, 2011; Ludick and Figley, 2017; Molnar et al., 2017; Sprang et al., 2019). It seems legitimate to hypothesize that such induced stress could impair task performances while in VR. Proper training and desensitization with time may reduce risks for workers to present Secondary Traumatic Stress and cope with it: e.g. police workers (Perez et al., 2010; Fortune et al., 2017; Grant et al., 2019). However, while working in VR, distressing material might induce acute stress workers need to cope with while performing tasks	Allow users to control exposure to distressing materials by applying filters on images, videos (Perez et al., 2010)
S_9 *IV*	Noise	In an office, we can speculate the noise is intermittent (Reinten et al., 2017): speech, phones ringing, software sound design, typing, printing, and walking sounds. These noises contribute to stress at the workplace (Jahncke and Hallman, 2020). Background noise in an office and conversation ranges from 50 to 70 dB (Abouee-Mehrizi et al., 2020). Irrelevant speech noises to a given task and unpredictability impair task performance (Szalma and Hancock, 2011; Marsh et al., 2018; Vasilev et al., 2018). Noise contributes to distraction and disturbance (Vasilev et al., 2018; Abbasi et al., 2020; Jahncke and Hallman, 2020; Minutillo et al., 2021). Noise in a shared VR environment could distract and disturb work (Zeroth et al., 2019)	Create sound control options for users to create a quiet environment. Reduce interface sound feedback, other users' conversations in a collaborative environment (Zeroth et al., 2019)

Depending on the tasks at hand, the interactions, and the relevant interfaces, acute stress in VR can arise accordingly. Just considering the possibility of stress while using VR may already help create safe working conditions and promote more benevolent work conditions. VR allows for teleporting users to a stress-relieving environment [natural surrounds (e.g., trees, grass, indoor biophilic environment) as well as light conditions (Van den Berg et al., 2015; Liu M. Y. et al., 2017; Yin et al., 2018; Hedblom et al., 2019; Wang et al., 2019; Huang et al., 2020; Kerous et al., 2020; Li C. et al., 2020; Park et al., 2020; Shuda et al., 2020; Li et al., 2021), music (Sokhadze, 2007; Nakajima et al., 2016; Yu C. P. et al., 2018; Paszkiel et al., 2020; Yin et al., 2020)]; and could help alleviate the above-described symptoms via this capacity (Thoma et al., 2013).

### 3.5. Mental overload

#### 3.5.1. Definition

Mental workload can be defined as “*a subjectively experienced physiological processing state, revealing the interplay between one's limited and multidimensional cognitive resources and the cognitive work demands being exposed to*” (Young et al., 2015; Ahmaniemi et al., 2017; de Witte et al., 2020) indicated that overload “*occurs […] when the operator is faced with more stimuli than (s)he is able to handle while maintaining their own standards of performance.”*

#### 3.5.2. Prevalence

Current knowledge does not allow us to define mental overload prevalence induced by VR use specifically outside of wild task-specific aspects. But, mental fatigue appears to be higher in VR as compared to conducting the same tasks in real offices (Van Acker et al., 2018). Furthermore, VR induces a higher mental workload than PC (Lim et al., 2013; Zhang et al., 2017; Broucke and Deligiannis, 2019; Makransky et al., 2019). But, contradictory results regarding mental workload have been observed (Porcino et al., 2017). For example, VR presents a lower cognitive demand for geo-visualization and trajectory data exploration than PC usage (Collaboration, 2015; Kaplan et al., 2020; Szopa and Soares, 2021), and a higher mental workload does not always negatively impact task performance (Tian et al., 2021). As mental overload is especially contingent on task characteristics, relying only on a general model provides only general assertions. Examples exist in air traffic control (Young et al., 2015), driving (Paxion et al., 2014; Tobaruela et al., 2014), as well as work in nuclear power plants (Wickens, 2017). Therefore, we here consider primarily two factors (general enough to apply to a wide variety of tasks). However, (Wickens, 2017) have previously considered 26 factors that could influence mental workload. In VR, task characteristics impact mental workload, via interactions and interfaces. We thus focus especially on time pressure and task difficulty.

#### 3.5.3. Guidelines considering mental overload factors

Based on our present synthesis of previous works, Table 15 features time pressure and task difficulty as these are the main factors influencing mental overload.

**Table 15:** Guidelines for two possible factors influencing mental overload.

**ID_factor Evidence level**	**Factor**	**Description**	**Guidelines**
MO_1 *VII*	Time pressure	Time pressure is associated with a higher mental workload (Hendy et al., 1997; Wang et al., 2016) and negatively affects task performance (Palada et al., 2018; Rieger et al., 2021) See also S_5	Consider giving more time to fulfill tasks in VR than on PC Try to measure the ideal (required) time necessary for a task to avoid imposing irrelevant time pressure (Liu and Li, 2020) Give a deadline for a task See also S_5
MO_2 *V*	Task difficulty	See also S_6 Basic interactions and interfaces can influence task difficulty (Yan et al., 2017; Geiger et al., 2018; Speicher et al., 2018; Zielasko et al., 2019; Biener et al., 2020; Gao et al., 2021; Wagner et al., 2021; Wu et al., 2021). Spatialization within VR seems to reduce mental workload only if tasks require such cognitively-related resources (Filho et al., 2018, 2020; Wismer et al., 2018; Armougum et al., 2019; Bernard et al., 2019; Broucke and Deligiannis, 2019; Baceviciute et al., 2021) Multitasking (Ahmad et al., 2021), especially interruptions (Cheng et al., 2020; Mcmullan et al., 2021) impacts negatively performance due to higher mental workload. Incongruent (with the primary task) emails (Addas and Pinsonneault, 2018), notifications (Tan et al., 2020) distract users	Consider reducing tasks' difficulty by: Reducing multitasking (fewer notifications, no incongruent emails during a given task) and allow users to predict multitasking (Ewolds et al., 2021) Testing interactions and interfaces to make sure they do not require unnecessary working memory solicitations by using questionnaires such as the NASA-TLX (Hart and Staveland, 1988; Hart, 2006; Grier, 2015; Hertzum, 2021) can be used only using spatialized information and interaction if the tasks require it Provide virtual assistant, visual cues, and feedback on how users are fulfilling tasks and their mental workload to help them focus on the primary task (Weng et al., 2017; Borghouts et al., 2020) Consider adapting interactions and interfaces based on the user's characteristics or preferences (Chen et al., 2019) In collaboration requiring object localization by speaking, avoid the spatial configurations diagonally in front and behind speakers (Milleville-Pennel et al., 2020) Allow users to train enough at tasks, interactions, and interfaces See also S_6

## 4. Discussion and limitations

We have provided a review featuring human factors and ergonomic approaches that have considered 90 factors that are proposed as impacting VRISE. More particularly, we considered 50 factors related to cybersickness in VR. Additionally, we examined fourteen factors involved with visual fatigue in VR and 15 related to muscle fatigue and musculoskeletal discomfort in VR. Finally, we identified nine factors for acute stress when working in VR, alongside two factors critical for mental overload assessment in VR.

General guidelines that designers should follow for a healthy, safe, and performant user experience at work:

- Design environments such that users can fulfill most of their tasks within 20-min interval to reduce cybersickness and visual fatigue occurrence.
- Provide an “exploration phase,” so that users can preview the fundamentals of their interactions, as well as experiencing local system feedback to reduce cybersickness and mental overload occurrence.
- Provide the user with a virtual assistant to adapt both interactions and interfaces to reduce mental overload occurrence.
- Limit movements within the virtual environment and display stereoscopy only when tasks require explicit depth cues to reduce cybersickness and visual fatigue occurrence.
- Create display features by considering user is sitting but allowing them to stand and walk on occasion to reduce muscular fatigue and musculoskeletal discomfort occurrence.
- Emphasize teleportation with guides for orientation if re-location within the virtual environment is necessary to reduce cybersickness.
- Allow users to customize their experience in the virtual environment (e.g., avatar, interface, and interactions) to reduce cybersickness, mental overload, and acute stress occurrence.
- Provide a monitoring toolkit that is based on questionnaires and psychophysiological measures, which allows to determine a user's susceptibility to side effects and to detect while they are immersed to reduce all VRISE occurrence.
- Provide stress-relieving procedures: these include, but are not limited to, nature (trees, grass, indoor biophilic environment), daylight, and relaxing music to reduce acute stress occurrence.

General guidelines that employers should follow for a healthy, safe, and effective use of virtual environments:

- Train workers to employ hardware and software effectively. This allows habituation and desensitization for the riskiest populations regarding cybersickness, reduces technostress that can provoke acute stress, and promotes an optimal degree of mental workload to reduce mental overload occurrence.
- Rethink and recast working tasks such that they can be readily adapted to virtual environments and their constraints to reduce acute stress and mental overload occurrence.
- Monitor workers' psychophysiological reactions in the virtual environment to record data to establish use benefit/risk ratios to reduce each VRISE occurrence.
- Have workers fill out anonymous questionnaires that inform about their individual susceptibility to VRISE.

General guidelines that workers would be informed of to sustain a healthy, safe, and effective use of virtual environments:

- Cease using virtual environments when symptoms of cybersickness, visual fatigue, muscle fatigue, and stress are experienced or task performance breakdowns occur.
- Take breaks following the use of virtual environments (take micro-naps, where possible walk beyond the bounds of the workplace, go drink water, seek “natural” spaces, listen to relaxing music or any and all combinations thereof) to reduce all VRISE symptoms.
- For those beyond 40 years of age, consider the individual to be might be more susceptible to elements of these side effects.
- Those with pathologies and/or particularities (e.g., eye diseases, overweight, neuroatypical, epilepsy, balance issues, muscle issues, and cognitive particularities), should be considered more susceptible to specific side effects of virtual environments.

Some prior guidelines have been suggested for discrete factors to promote healthier, safer, and more efficient work with virtual environments (Gabbard et al., 1999; Stanney et al., 2003b, 2021b; Burkhardt et al., 2006; Bando et al., 2012; Lanier et al., 2019; Muthukrishna and Henrich, 2019; Chen et al., 2021). However, most of these works concentrated on only one VRISE at a time. Frequently, they are not clear on the level of confidence associated with each guideline. However, to build on these previous works, we categorized factors into three types: individual, hardware, and software. With our tables, readers and stakeholders can easily refer to the present work as a guide for their design or use of virtual environments. Hence, the present offering is the most substantial and comprehensive assessment for the VR community. This is because it encompasses the greatest assemblage of information while providing the most practical and useful survey and recommendations.

The occurrence of acute stress and mental overload can be influenced by many further factors than those presented in our guidelines. Moreover, the factors and associated guidelines for all five VRISE are based on current knowledge. Further theoretical and experimental contributions are still needed to explain VRISE better by encompassing its inherent complexity. We must be aware that some factors are similar across VRISE (Souchet et al., 2022). We present them for each short-term VRISE to emphasize those similarities and better demonstrate confounding effects that remain to be addressed.

Some guidelines do not apply to all workers as we purposely selected only office-like tasks to contextualize our current contribution to the ergonomics of VR. However, very few existing works have been directed at tackling VRISE. Currently, the primary uses of VR lie in video games (entertainment in general) and training (see Cockburn et al., 2020). Consequently, our guidelines are sometimes based on observations, not directly on experiments using virtual environments for work or VR. Part of our guidelines still rests upon low evidence. Cybersickness is the VRISE with the most robust evidentiary basis. However, most meta-analyses, as well as systematic reviews, are founded upon questionnaire responses. Questionnaires appear to be the most utilized approach for all VRISE. Therefore, confidence in tested techniques to reduce VRISE relies, to the present time, less on objective measurements than might be preferred (Souchet et al., 2022).

Moreover, experimental quality and reproducibility need improvement in the VR field, which is valid for psychology and human-computer interaction in general (Chang et al., 2020; Petri et al., 2020; Gilbert et al., 2021; Halbig and Latoschik, 2021; Biener et al., 2022). Therefore, designers, employers, and workers should be cognizant that some factors tackled here and the associated guidelines are sometimes a direct transposition from the scientific literature that has not directly tackled VRISE or the work context. Such literature might suffer from shortcomings. However, it also means that part of the guidelines can be generalized to other contexts than work: i.e., entertainment and skills training. The median evidence level crystallizes this: five for cybersickness, four for visual fatigue, six for muscular fatigue, five for stress, and six for mental overload. We applied a scale from the medical field which hasn't been created for ergonomics issues, and proof that it is entirely relevant in this very case is low. Mainly because most scientific experiments in VR very rarely follow a large multisite randomized controlled trial methodology.

One major limitation of this study is that we concentrated on short-term VRISE. However, working in VR implies daily use, and a pre-print (Biener et al., 2022) documented VR work for one week. VR appears to be worse than PC working. Cybersickness is a concern, and some participants even dropped out of the study. The advantages and disadvantages of VR's long-term use are yet to be drawn. Following the present guidelines might help foster advantages, but they cannot delete disadvantages.

Another major limitation of our contribution is the included papers. We stopped inclusion in the review with papers published in mid-2021. However, several relevant papers were published at the end of 2021, in 2022, and at the beginning of 2023. Those relevant publications include guidelines for each VRISE, side effects mitigation technics, prediction and detection of side effects. This fosters the need for the research community to critique and update these guidelines.

Future valuable contributions regarding VRISE factors and guidelines to reduce any such impacts include the following:

1) Increasing experimental contributions testing influences of each factor on VRISE with high-quality methods using within-subject, between-subject, and crossover designs,
2) Increasing considered VRISE to allow a better risk/benefit ratio consideration to use VR or not,
3) Increasing experimental contributions regarding tangles between VRISE,
4) Advancing automatic VRISE detection based on psychophysiological measurements,
5) Contributing to publications looking at the big picture of VR via systematic reviews and meta-analysis,
6) Updating the current guidelines with stronger evidence.

Although important to follow our guidelines, stakeholders should remain aware that current HMDs and virtual environments will most likely induce cybersickness, visual fatigue, muscle fatigue, acute stress, and mental overload. Currently, no existing method can fully alleviate these VR side effects. Therefore, detecting and adapting the virtual environment based on psychophysiological measurements (Smith and Du'Mont, 2009) could help better individualize and optimize the user experience. A better understanding of all VRISE risks will allow a benefit/risk ratio assessment to decide when to use virtual environments or not.

## Author contributions

AS, DL, J-MB, and PH contributed to conception and design of the review. AS wrote the first draft of the manuscript. All authors contributed to manuscript revision, read, and approved the submitted version.

## Footnotes

^1^Due to the current limitations to 32 words, two requests were done distributing between the Former and latter VRISE.
