# Corrigendum: Design guidelines for limiting and eliminating virtual reality-induced symptoms and effects at work: a comprehensive, factor-oriented review

**DOI:** 10.3389/fpsyg.2023.1261103

**Published:** 2023-08-21

**Authors:** Alexis D. Souchet, Domitile Lourdeaux, Jean-Marie Burkhardt, Peter A. Hancock

**Affiliations:** ^1^Heudiasyc UMR 7253, Alliance Sorbonne Université, Université de Technologie de Compiègne, CNRS, Compiègne, France; ^2^Institute for Creative Technologies, University of Southern California, Los Angeles, CA, United States; ^3^IFSTTAR, University Gustave Eiffel and University of Paris, Paris, France; ^4^Department of Psychology, University of Central Florida, Orlando, FL, United States

**Keywords:** virtual reality, ergonomics, cybersickness, visual fatigue, muscle fatigue, acute stress, mental overload, work

In the published article, there was an error regarding the affiliation for author Domitile Lourdeaux. Their affiliation was stated as “2 Institute for Creative Technologies, University of Southern California, Los Angeles, CA, United States” but should be “1 Heudiasyc UMR 7253, Alliance Sorbonne Université, Université de Technologie de Compiègne, CNRS, Compiègne, France”.

In the published article, there was also an error in Discussion and Limitations, Paragraph 9. The second sentence cited “(Rebenitsch and Owen, 2017)” but it should be “(Biener et al., 2022)”. The corrected paragraph appears below:

One major limitation of this study is that we concentrated on short-term VRISE. However, working in VR implies daily use, and a pre-print (Biener et al., 2022) documented VR work for 1 week. VR appears to be worse than PC working. Cybersickness is a concern, and some participants even dropped out of the study. The advantages and disadvantages of VR's long-term use are yet to be drawn. Following the present guidelines might help foster advantages, but they cannot delete disadvantages.

The authors apologize for these errors and state that this does not change the scientific conclusions of the article in any way. The original article has been updated.
